# Effect of Different Levels of Chlorogenic Acid on Growth Performance, Immunological Responses, Antioxidant Defense, and Disease Resistance of Rainbow Trout (*Oncorhynchus mykiss*) Juveniles

**DOI:** 10.1155/2023/3679002

**Published:** 2023-04-19

**Authors:** Hamed Ghafarifarsani, Shiva Nedaei, Seyed Hossein Hoseinifar, Hien Van Doan

**Affiliations:** ^1^Department of Fisheries, Faculty of Natural Resources, Urmia University, Urmia, Iran; ^2^Department of Fisheries Science, Faculty of Marine Science and Technology, University of Hormozgan, Bandar Abbas, Iran; ^3^Department of Fisheries, Faculty of Fisheries and Environmental Sciences Gorgan University of Agricultural Sciences and Natural Resources, Gorgan, Iran; ^4^Department of Animal and Aquatic Sciences, Faculty of Agriculture, Chiang Mai University, Chiang Mai, Thailand

## Abstract

The current study is designed to assay the efficacy of chlorogenic acid (ChA) in the diet on growth performance, digestive enzyme activity, serum immunological, biochemical, and antioxidant variables, and mucosal immune response as well as disease resistance of rainbow trout (*Oncorhynchus mykiss*) juveniles. Rainbow trout juveniles received diets supplemented with different inclusion levels of ChA (0 (ctrl), 200 (CA1), 400 (CA2), 600 (CA3), and 800 (CA4) mg kg^−1^ diet) for 60 days. According to the findings, fish from CA3 and CA4 groups demonstrated the best results considering the final weight (FW) and weight gain (WG) (*P* < 0.05). Also, the group that received 600 mg kg^−1^ ChA-supplemented diet showed the lowest feed conversion ratio (FCR) and the highest specific growth rate (SGR) compared to other groups (*P* < 0.05). Moreover, the minimum survival rate (SR) was only detected in the CA4 treatment (*P* < 0.05). Regression analysis exhibited that rainbow trout growth indices were polynomially linked to dietary chlorogenic acid concentrations. In this regard, the optimal levels of chlorogenic acid according to growth parameters (FCR and SGR) were 0.71 and 0.62 gr kg^−1^ diet, respectively. The results exhibited superior performance of protease and amylase activities in CA2, CA3, and CA4 groups with the maximum amount in the group receiving 600 mg kg^−1^ ChA-enriched diet (*P* < 0.05). Serum lysozyme (LYZ), immunoglobulin (Ig), and components 3 and 4 (C3 and C4) values of CA2, CA3, and CA4 groups were significantly higher than others with the highest amount in the CA3 group (P <0.05). Additionally, serum nitroblue tetrazolium (NBT) value in the CA3 and CA4 groups and myeloperoxidase (MPO) in the CA3 group were notably more than others (*P* < 0.05). Moreover, the lowest aspartate aminotransferase (AST), alkaline phosphatase (ALP), alanine aminotransferase (ALT), and lactate dehydrogenase (LDH) and the highest total protein (TP) and globulin (GLO) values were observed in CA3 treatment (*P* < 0.05). CA2 and CA3 groups demonstrated increased serum catalase (CAT) and decreased malondialdehyde (MDA) values compared to the control while the highest CAT and lowest MDA values were observed in CA3 treatment (*P* < 0.05). Considering mucus immunity, the significantly maximum LYZ and protease values were demonstrated in CA2 and CA3 groups, and the highest ALP, Ig, and esterase values were demonstrated in the CA3 group. In comparison with the control, the mortality rates of the groups that received the ChA diets were remarkably (*P* < 0.05) lower postchallenge with *Y. ruckeri*, and the highest survival and relative percentage of survival (RPS) (*P* < 0.05) belonged to the CA3 group. Results obtained from the current study suggested ChA as a functional dietary additive to raise growth parameters, immune indices, antioxidant capacity, and resistance to disease in rainbow trout.

## 1. Introduction

Fish farming systems are usually exposed to various pathogens and diseases due to the high density of fish [1]. Currently, a variety of chemicals such as antibiotics and antifungal chemicals are used to control and treat diseases in fish farms [2, 3]. Moreover, various vaccines have been proposed to immunize and treat viral diseases; however, these vaccines have not usually shown high efficiency in treatment of viral diseases [4, 5]. For bacterial diseases, applying antibiotics in fish farms is associated with environmental restrictions and concerns [3]. Accumulation of antibiotics and metabolites resulting from their metabolism may threaten human health. In addition, the continuous application of antibiotics may make resistant strains [3, 6]. Release of antibiotics into nature from the outlet water of the pools may inappropriately change the natural fauna and flora of aquatic ecosystems [7, 8]. Because of these limitations and concerns, replacing antibiotics with safe and efficient substances can be a useful solution. In this regard, applying immunogenic supplements can be a useful way to enhance fish immunity and resistance to pathogens [9–12]. A wide range of natural and synthetic immunogenic supplements have been successfully used in the diet of fish and have resulted in improved growth and immunity [13].

Medicinal plants and compounds derived from them are among the most common immunogenic supplements examined in aquaculture [14–16]. The biochemical composition of medicinal plants includes compounds with immunogenic and antioxidant properties such as terpenes, flavonoids, and phenols [17–20].

Chlorogenic acid (ChA) is a well-known phenolic compound naturally found in a wide range of herbs, such as coffee beans, sunflower, eucommia, and honeysuckle [21, 22]. ChA has shown various biological properties including immunogenic, antibacterial, antioxidant, and anti-inflammatory actions [21–25]. Therefore, these properties may indicate the potential of ChA in improving the health and immune system of animals. In few studies on fish and shellfish, it has been recognized that ChA prompts growth and immunity [22, 26–28]. For example, in grass carp, ChA has dietary levels of 0.01–0.06% [26], 400 mg ChA/kg diet [29, 30]. However, 200–800 mg/kg diet had no significant effect on growth performance of Koi, *Cyprinus carpio* [22]. In addition, it was recognized that ChA can enhance immunity and antioxidant defense in grass carp and Koi [22, 31] and also in largemouth bass, *Micropterus salmoides* [32]. However, there is no data available regarding the protective role of ChA against fish pathogens, especially in commercial fish species.

Rainbow trout, *Oncorhynchus mykiss*, is well known as the most important cold water-farmed fish of the family Salmonidae throughout the world [33, 34]. Trout farms are usually exposed to epidemics of various opportunistic bacterial diseases due to the high density of the fish and sometimes poor management of wastes [35]. *Yersinia ruckeri* is an opportunistic bacteria and the agent of yersiniosis or enteric redmouth diseases in rainbow trout [36]. Reports have shown that losses caused by *Y. ruckeri* can be up to 70% [37]. In this study, the potentials of ChA were investigated on growth, immunological responses, antioxidant defense, and resistance to *Y. ruckeri* infection in the rainbow trout (*O. mykiss*). The results of this study may suggest an environmentally healthy way to control the infection in rainbow trout farming.

## 2. Materials and Methods

### 2.1. Experimental Diets

A control diet was formulated by Lindo feed formulation software according to rainbow trout nutrient needs (Table 1) as suggested by Yousefi et al. [38]. The proximate composition of the basal diet was defined considering AOAC methods [39]. In this study, chlorogenic acid (ChA) (purity > = 95%) was provided by Sigma-Aldrich. Different levels of ChA (0, 200, 400, 600, and 800 mg/kg) were incorporated with a basal diet, through the equal decrease of wheat meal concentration at each level, to have five experimental diets, named C (control), CA1, CA2, CA3, and CA4, respectively, as recommended dose of chlorogenic acid applied by Xu et al.[22] previously. The feedstuffs were combined, and subsequently, enough distilled water was used to have a dough and next passed through an industrial meat grinder. The pellets were retained at room temperature for 36 h and subsequently maintained in plastic bags at 4°C for further evaluation.

### 2.2. Experimental Design

For this study, a total of 600 rainbow trout juveniles (12.20 ± 0.3 g; mean ± SE) were obtained from a private local farm (Yasuj, Kohgiluyeh and Boyer-Ahmad province) and transferred to the laboratory setting (Karaj, Iran). All fish were permitted to adapt to the lab setup in fiberglass tank (1000 L) at 15°C for two weeks and received a control (unsupplemented) diet *ad libitum*, three times per day within this stage. Afterward, 525 fish (18.17 ± 0.05 g; mean ± SE) were accidentally assigned to 15 tanks (35 samples per tank, total volume 300 L, water volume 150 L) to design an experiment with 5 experimental groups triplicate each, including C (control), CA1, CA2, CA3, and CA4. During the rearing period (60 days), fish were hand fed thrice a day (8:00 am, 1:00 pm, and 7:00 pm) up to apparent satiety and the biomass of every replicate was calculated every 14 days to modify the feed quantity. 4 h post feeding, the remaining pellets at the bottom of every tank were gathered, dried at 60°C, and then deducted from the given food. All tanks were aerated continuously by an air pump. Daily water exchange (70%) with aerated, fresh, and dechlorinated water was done, and suspended particles were eliminated manually by siphoning every day throughout the experiment. The culture water physicochemical parameters such as temperature 15.50 ± 0.5, DO 8.00 ± 0.2 mg/L, pH 7.3 ± 0.18, and nonionized ammonia 0.03 ± 0.005 were monitored during the feeding trial.

### 2.3. Growth Indices

After the rearing period, 24 h post last feeding, fish were anesthetized with clove essential oil (100 mg/L); next, all fish were weighed to determine growth performance parameters using the formulas below [38].

(1) $$\begin{array}{c} \text{Weight gain}\left(\text{WG},\text{g}\right)=\text{final weight}–\text{initial weight},\\ \text{Feed conversion ratio}\left(\text{FCR}\right)=\frac{\text{feed intake}\left(\text{g}\right)}{\left(\text{final weight}–\text{initial weight}\right)},\\ \text{Specific growth rate}\left(\text{SGR},\%/\text{d}\right)=100\times \left[\frac{\left(\ln\text{ final weight}-\ln\text{ initial weight}\right)}{\text{days}}\right],\text{Survival rate}\left(\text{SR},\%\right)=100\times \left(\frac{\text{final number}}{\text{initial number}}\right). \end{array}$$

### 2.4. Sampling Procedure

To evaluate serum immunological parameters, feeding was stopped for 24 h. Then, a 2 mL syringe was applied to obtain blood samples from the caudal vein of three anesthetized fish per replicate and the supernatant was disassembled after centrifugation (3000*g* for 10 min at 4°C). The acquired serum was then kept at −70°C for future evaluation [40]. Also, to evaluate skin mucus parameters, three fish per replicate randomly were placed in plastic bags containing 10 mL of 50 mM physiological saline. After three minutes, the obtained mucus was centrifuged (2500*g* for 10 min at 4°C) and the supernatant was kept at −80°C for further analysis [41]. To evaluate digestive enzyme activities, three anesthetized fish were killed; next, the entire intestine was collected, emptied, and rinsed with saline solution (50 mM) and then homogenized using Tris buffer (Heidolph® SilentCrusher M, Heidolph, Nuremberg, Germany). The mixture was centrifuged (6000*g*) for 10 min at 4°C; thereafter, the supernatant was maintained at −80°C until evaluation.

#### 2.4.1. Digestive Enzyme Activities

Amylase activity was estimated based on Robyt and Whelan [42] applying 2% starch as a substrate in 500 microliters of 0.1 M phosphate buffer. Lipase enzyme activity was measured in agreement with Iijima et al. [43], applying p-nitrophenyl myristate in cholate buffer (0.25 mM Tris HCl + 0.25 mM 2-methoxyethanol + 5 mM sodium cholate, pH = 9.0). The mixture was incubated (15 min, 30°C) and paused by acetone/n-heptane (5 : 2, *v*/*v*). The absorbance of the supernatant was read at 405 nm. Total protease value was measured in the manner of García-Carreño [44] applying azocasein in 0.5 mL Tris (0.1 M Tris-HCl; pH = 8) and incubated applying 5% trichloroacetic acid at 25°C for 1 h. After the centrifugation of the mixture, the absorbance of the supernatant was recorded at 440 nm.

#### 2.4.2. Serum Antioxidant Enzyme Activities

Superoxide dismutase (SOD) activity was calculated using commercial kits (Zellbio, Hamburg, Germany) and conversion of superoxide anion to hydrogen peroxide protocol [45]. The value of SOD needed to inhibit the rate of reduction of cytochrome C by 50% was considered as 1 unit of SOD activity. Catalase (CAT) enzyme activity was specified according to the decomposition of hydrogen peroxidase per minute as described by Goth [46]. One unit of CAT activity was considered as the value of enzyme needed to break down 1 *μ*mol of H_2_O_2_ per min. The glutathione peroxidase (GPx) activity was estimated by the transformation of glutathione to glutathione disulfide as reported by Hoseini et al. [45]. The amount of malondialdehyde (MDA) was estimated considering the reaction with thiobarbituric acid at 95°C. All parameters were calculated utilizing commercially available kits (Zellbio, Hamburg, Germany).

#### 2.4.3. Serum Biochemical Indices

Evaluation of alkaline phosphatase (ALP), aspartate aminotransferase (AST), alanine aminotransferase (ALT), and lactate dehydrogenase (LDH) was estimated utilizing available kits (Pars Azmun, Co., Tehran, Iran), in the manner of manufacturer's procedure [47]. Total protein (TP) was calculated conforming to Bradford [48], applying bovine serum albumin as standard. Albumin (ALB) was detected colorimetrically at 620 nm as described by Nicholson [49], and globulin (GLO) content was measured by subtracting the TP and albumin contents.

#### 2.4.4. Nonspecific Immunity Assay

*(1) Serum and Mucus Immune Parameters*. Lysozyme (LYZ) activity was evaluated by turbidimetric assay as suggested by Ellis [50], applying *Micrococcus lysodeikticus* (Sigma) as a target bacteria. Total immunoglobulin (Ig) was calculated according to the protein content before and post addition of polyethylene glycol.

*(2) Serum Immune Parameters*. The complement components (C3 and C4) of serum were evaluated by an ELISA device (ELX800, BioTek, Vermont, USA) utilizing an available kit (Pars Azmun Co., Tehran, Iran). The alternative complement activity (ACH50) was calculated according to Yano [51]. In this method, sheep red blood cells in vernal tissue containing EGTA and manganese were considered as targets. Thereafter, different concentrations of serum samples (10, 5, 2.5, 1.25, 0.625, and 0.312%) were prepared and 25 microliters of each sample was mixed with 125 *μ*L of buffer and 50 *μ*L of blood cells. Then, 2 hours after incubation at room temperature, the mixture was centrifuged and the absorbance was recorded at 412 nm. Serum nitroblue tetrazolium (NBT) was assayed based on Anderson and Siwicki [52] method using N,N-dimethylformamide. Briefly, 100 *μ*L of heparinized blood was added to 100 *μ*L of 0.2% NBT. The combination was incubated for 30 min at 25°C. Then, 50 *μ*L of the obtained suspension was added to 1 mL of N,N-dimethylformamide and centrifuged at 3000 for 5 min, and the absorbance of the upper solution was recorded at 540 nm. Myeloperoxidase (MPO) activity was calculated as reported by Quade and Roth [53]. In this method, 10 *μ*L of serum sample in combination with 90 microliters of Hank's balanced salt solution (HBSS) without cerium and magnesium ions was added to 96-well pellets. Then, 35 *μ*L of tetramethylbenzidine hydrochloride (TMB) system was added too. The color change was stopped by adding 35 *μ*L of 0.5 M sulfuric acid, and the absorbance was recorded at 450 nm.

*(3) Mucus Immune Parameters*. The protease activity was detected as reported by Hoseinifar et al. [54]. In brief, an equal volume of the sample was combined with 100 microliters of ammonium bicarbonate buffer containing 0.7% azocasein solution. The mixture was incubated at 30°C for 20 h and finished utilizing trichloroacetic acid. Thereafter, the supernatant was collected after centrifugation (15000*g* for 5 min). Then, the mixture was combined with 5.5 normal hydroxide and the absorbance was read at 450 nm. ALP activity was measured by the available kit (Pars Azmun, Co., Tehran, Iran) and considering the manufacturer's protocol [55]. Esterase value was calculated according to Guardiola [56]. For this, 0.4 mM mucus and 0.4 mM p-nitrophenyl myristate as substrate were incubated in ammonium bicarbonate buffer containing 0.5% Triton X-100 at 30°C and the absorbance was read at 405 nm.

### 2.5. Challenge Test

The bacterial challenge was carried out in a place away from the culture environment for 14 days, using *Yersinia ruckeri* bacteria (KC291153) considering restrict quarantine and hygiene protocols. For this purpose, *Y. ruckeri* was incubated in a broth culture medium for 48 h at 27°C. Eventually, following the growth phase, the bacterial suspension was centrifuged and the bacterial stock was rinsed double by phosphate buffer. Bacterial concentration was adjusted by serial dilution at 10^7^ cells per mm [57]. After the culture period, 15 samples per replicate were injected with 0.1 mL of the bacterial suspension. The mortality rate was measured throughout the period. Also, 2 weeks after the challenge, the mortality rate and relative percentage of survival (RPS) were calculated [58].

(2) $$\begin{array}{c} \text{RPS}\left(\%\right)=\left(1-\frac{\%\text{of mortality in treated groups}}{\%\text{of mortality in the control group}}\right)\times 100. \end{array}$$

### 2.6. Statistical Analysis

This experiment was done in the form of a completely randomized design. For this, the normality of the obtained data was assessed using the Kolmogorov-Smirnov test. Statistical investigation of the data was done by SPSS 20 software and with the help of one-way ANOVA and Tukey's HSD test. Broken-line regression model was applied to specify the optimal level of ChA according to the measured parameters. It is worth mentioning that all the tests were interpreted at a significance level of less than 5% and the final results were reported as mean ± SE. Excel version 2013 software was used to draw graphs.

## 3. Results

### 3.1. Growth Rate

**Figure 1:**
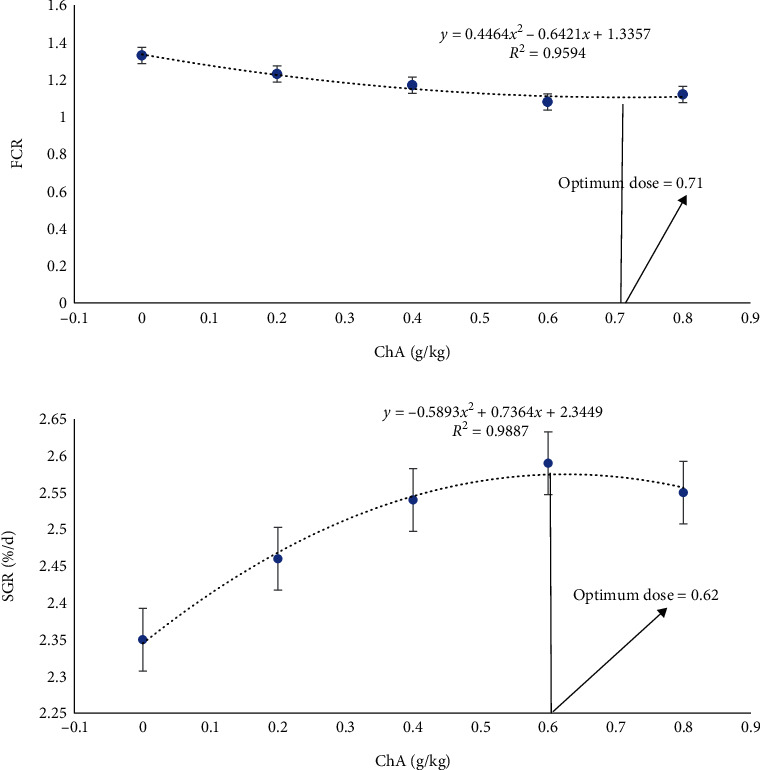
Relationships between the dietary ChA levels and growth parameters of rainbow trout (*n* = 3).

Table 2 represents the results of dietary ChA supplementation concerning the growth parameters. Based on findings, ChA-supplemented diets were significantly efficient to improve growth performance parameters (FW, WG, SGR, and FCR). Indeed, the lowest growth performance belonged to the treatment that received basal diet (*P* < 0.05). The maximum FW and WG amounts were detected in CA3 and CA4 treatments (*P* < 0.05) with no notable dissimilarity between these treatments. Also, the groups that received CA2, CA3, and CA4 diets showed significantly lower FCR in comparison with those that received basal diet (*P* < 0.05). Additionally, fish from the CA3 group showed the best result considering SGR and FCR as opposed to other treatments (*P* < 0.05). Also, the minimum SR amount was only detected in the CA4 group (*P* < 0.05). For growth immune parameters, 0.71 and 0.62 gr kg^−1^ ChA diet were chosen as the optimum dose of chlorogenic acid for FCR and SGR, respectively, by the broken-line regression analysis (Figure 1).

### 3.2. Digestive Enzyme Activity

**Figure 2:**
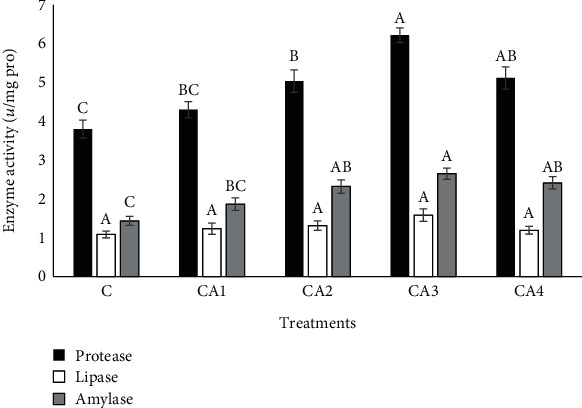
Digestive enzyme activity of *O. mykiss* fed with different levels of ChA for 60 days. Columns assigned with different superscripts significantly differ (*P* < 0.05). Values are presented as mean ± SE.

The digestive enzyme activities of fish that received multiple concentrations of dietary ChA are presented in Figure 2. The results showed the better performance in terms of protease and amylase contents in CA2, CA3, and CA4 groups in comparison with the unsupplemented group with the maximum amount at CA3 treatment (*P* < 0.05). However, diets containing ChA could not successfully change the activity of lipase contrasted to the control treatment (*P* < 0.05).

### 3.3. Serum Immunological Response

Table 3 shows the efficacy of ChA on serum immune parameters. Serum LYZ, Ig, C3, and C4 levels of the CA2, CA3, and CA4 treatments were remarkably more than those of the unsupplemented treatment with the highest value in the CA3 group (*P* < 0.05), although no considerable dissimilarity was detected among CA1 and control treatments (*P* > 0.05). MPO content remarkably enhanced in the CA3 treatment in comparison with the unsupplemented treatment (*P* > 0.05), while no variance was found between other treatments (*P* < 0.05). Regarding ACH50, no considerable difference was demonstrated between control and ChA-supplemented treatments (*P* > 0.05). However, serum NBT values in the CA3 and CA4 groups were notably more than others (*P* < 0.05).

### 3.4. Mucus Immunological Response

The outcome of immune parameters in fish mucus is exhibited in Table 4. Based on the findings, the CA2 and CA3 groups demonstrated notably higher mucus LYZ activity in contrast to that of the unsupplemented treatment with no notable contrast between these treatments (*P* < 0.05). The significant difference in mucus ALP and Ig values was presented in CA2, CA3, and CA4 treatments (*P* < 0.05) with the maximum amount in the CA3 treatment. Protease content significantly improved in CA2, CA3, and CA4 treatments, while CA2 and CA3 showed the highest values compared to other treatments. Moreover, the CA3 group displayed higher esterase content in comparison with the unsupplemented treatment (*P* < 0.05).

### 3.5. Serum Biochemical Investigation

ChA-supplemented diets led to a decline in serum AST value at all inclusion levels. Additionally, ALP and ALT contents in CA2, CA3, and CA4 treatments and LDH in CA3 and CA4 treatments demonstrated lower amounts compared to the control (Table 5). However, the lowest level of AST, ALP, ALT, and LDH values was detected in CA3 treatment among others (*P* < 0.05). Serum TP levels of the CA2 and CA3 treatments were significantly more than those of the unsupplemented treatment and the maximum value detected in the CA3 treatment (*P* < 0.05). ALB value did not change among control and ChA-containing treatments (*P* > 0.05). However, considerable elevation in GLO value was only observed in CA3 treatment (*P* < 0.05).

### 3.6. Serum Antioxidant Status

**Figure 3:**
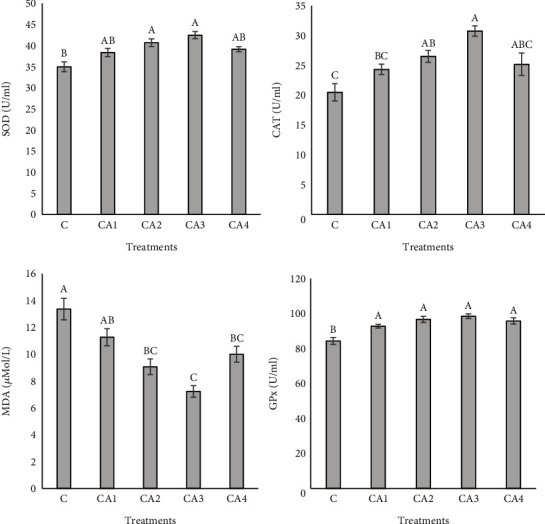
Serum antioxidant activity of *O. mykiss* fed with different levels of ChA for 60 days. Columns assigned with different superscripts significantly differ (*P* < 0.05). Values are presented as mean ± SE. CAT: catalase; SOD: superoxide dismutase; MDA: malondialdehyde; GPx: glutathione peroxidase.

The findings of serum antioxidant indices are demonstrated in Figure 3. Serum CAT activity of the CA2 and CA3 treatments was remarkably more than that of the control, and the maximum value was demonstrated in the CA3 treatment (*P* < 0.05). MDA value diminished significantly in CA2, CA3, and CA4 groups, while CA3 showed the lowest value among other groups. Additionally, the results revealed no significant variation in the case of the impact of ChA on SOD and GPx levels (*P* > 0.05).

### 3.7. Challenge with *Y. ruckeri*

**Figure 4:**
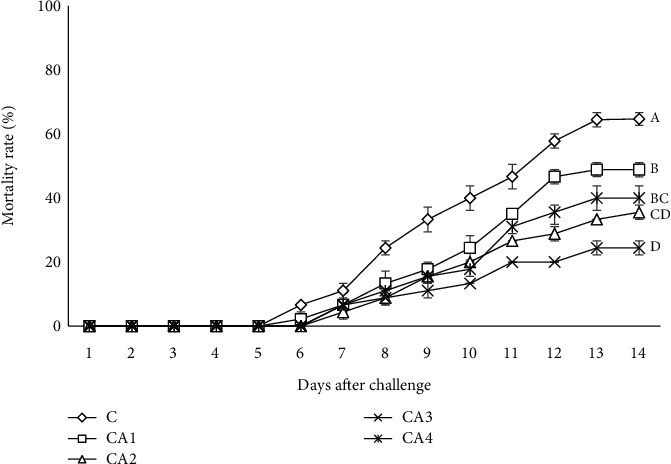
Mortality rate (%) of *O*. *mykiss* fed diets supplemented with different levels of ChA for 60 days after 14 days postchallenge with *Y*. *ruckeri*.

In comparison with the control, the mortality rates of groups that received ChA diets were remarkably (*P* < 0.05) lower postchallenge with *Y. ruckeri* (Figure 4), and the lowest amount was detected in the CA3 group. The findings of the challenge with *Y. ruckeri* exhibited that fish supplemented with ChA diets had notably higher survival than the unsupplemented group. The RPS of CA1, CA2, CA3, and CA4 groups were 24.47 ± 3.45, 45.04 ± 3.45, 62.25 ± 3.40, and 38.17 ± 5.92, respectively. Checking the data showed that the highest survival and RPS (*P* < 0.05) belonged to the CA3 group (Table 6).

## 4. Discussion

Growth, immune components, and resistance to diseases are among the most important parameters examined to evaluate the efficiency of dietary supplements in fish. The ChA is recognized to promote growth and immunity in domestic animals [24, 59–64]. However, very limited attention has been paid to ChA with fish. In this study, the use of dietary ChA improved the growth performance, especially in rainbow trout fed 600–800 mg ChA/kg diet. Although the growth-enhancing properties of ChA have been the focus of many studies in domestic animals, these studies are very limited in fish. In grass carp, ChA at dietary levels of 0.01–0.06% improved growth rate and FCR [26]. In the same species, Yang et al. [30] reported an improvement in the fish growth performance, following supplementation with a 400 mg ChA/kg diet. Sun et al. [29] observed improved growth in the grass carp, following supplementation with 400 mg ChA/kg diet. By contrast, ChA at a dietary level of 200–800 mg/kg diet had no significant effect on the growth performance of Koi, *Cyprinus carpio* [22]. Therefore, the effects of ChA on growth may be different depending on its levels in the diet and fish species. Although the mechanisms for growth-promoting effects of ChA are not yet known in fish, results from other animals have shown that ChA can improve growth performance by improving intestinal barrier functions [60, 61], immune parameters [28, 59], appetite, nutrient digestibility, protein biosynthesis [65], gut morphology, and microflora [66].

Based on our results, ChA had not much effect on the survival of the fish, although the survival was slightly lower in the 800 mg ChA/kg diet treatment. Owing to the enhanced immune and other health-related parameters of fish in this treatment, a reduction in growth could be unlikely. Anyway, the survival rate was above 90%, which is good and just a little lower than other treatments. Therefore, the lower survival rate in the 800 mg ChA/kg diet treatment may be due to factors other than the effect of ChA, such as individual differences with confinement and manipulation and the initial health status of the fish at the beginning of the experiment [67].

In the current study, we observed a considerable elevation in digestive enzyme activity, mostly in fish fed 600–800 mg ChA/kg diet. Therefore, the improved growth of ChA-treated fish may be due to the inducing effects of ChA on digestive enzymes. In general, the effect of ChA supplementation on digestive enzymes has not been studied yet, and even studies in other vertebrates are very limited. However, ChA as a bile diuretic compound may prevent bile accumulation in the gallbladder, thus improving the digestive system [68]. It is recognized that ChA decreases the accumulation of bile acids which are toxic for hepatocytes by downregulating the uptake transporters and bile acid synthetic enzymes and upregulating efflux transporters and bilirubin-conjugating enzymes.

Feeding fish with ChA enhances immune components of serum (TP, albumin, LYZ, Ig, C3, C4, ACH_50_, NBT, and MPO) and mucus (LYZ, Ig, protease, and esterase activities), especially in fish fed with 600–800 mg ChA/kg diet. Our results were in line with the results of other studies, where ChA improved immunity and antioxidant defense in domestic animals [24, 59, 63, 64] and fish [22, 31]. In the grass carp, supplementation of fish with 0.08% ChA significantly increased phagocytic activity and complement C3 concentration [31]. LYZ activity elevated in response to the 500–600 mg ChA/kg diet in Koi [22]. Total protein indicates the nutritional status of the fish and is also considered an indicator of the immune status, because the total protein includes antibodies and albumins [69]. The globulins are produced by the mononuclear phagocytes and have an important function of modulating the fish immune system [70]. In this study, ChA prompted total protein, albumin, and globulin content, in which these results indicate the immune-inducing effects of ChA in the fish.

As mentioned earlier, ChA is a phenolic compound and numerous studies have confirmed the improving effects of phenolic compounds on immunity and antioxidant defense in fish [71–75]. The antioxidant properties of the phenolic compounds are mainly returned to hydroxyl groups in their biochemical structures [76–78]. Oxidative stress in fish usually occurs in response to environmental stresses such as pollutants and pathogens, which can disrupt growth, immunity, and reproduction [79–82].

Enzymatic and nonenzymatic antioxidant defense system protects fish against oxidative stress and damage caused by FR [83]. Dietary ChA stimulated the activity of CAT, SOD, and GPx, mainly in fish fed 600–800 mg ChA/kg diet. Similarly, there are some studies reporting the role of ChA in enhancing the antioxidant capacity in fish and other animals [60, 61, 66, 84]. Li et al. [26] observed an increase in SOD activity, following supplementation with 0.04% ChA in the grass carp. The use of 500–600 mg ChA/kg in the diet of Koi stimulated the activity of SOD, GSH, and CAT [22]. In largemouth bass, *Micropterus salmoides*, ChA at a dietary level of 300–600 mg/kg diet induced the expression of SOD, CAT, and GPx genes [32]. In the current study, ChA showed an ameliorating function upon oxidative stress, especially in the treatments of 600–800 mg ChA/kg diet, as the levels of MDA were lower in these groups compared to unsupplemented fish.

MDA is generated upon peroxidation of lipids in the cell and thus is generally considered one of the main indicators of oxidative stress in fish and other vertebrates [27, 32, 60, 61, 85–88]. The ameliorating effects of ChA on oxidation stress may be due to its antioxidant properties, which can be attributed to the presence of phenolic functional groups in its biochemical composition [25, 89, 90].

The increased levels of liver metabolic enzymes (LMZ) in the blood are usually known as an indicator of liver disorders and damage, although this is a general assumption and not specific [91, 92]. In this study, ChA, especially at dietary levels of 600–800 mg ChA/kg diet, reduced the levels of LMZ, which may suggest a protective role for ChA in the liver. Although the protective effects of ChA on the fish liver have not been investigated yet, data from other animals have shown that ChA reduces the levels of liver enzymes in the blood, and in this sense, it may have a protective role on the liver [93–95].

As stated earlier, the main purpose of using immune stimulants in aquaculture is to increase resistance to diseases. In the current study, we challenged the fish with *Yersinia ruckeri* after the feeding period. After 14 days of challenge, the lowest mortality was observed in fish fed 600–800 mg ChA/kg diet, indicating therapeutic properties of ChA against *Y. ruckeri* infection. Although the effect of ChA on disease resistance in fish has not been examined yet, few studies using animals have shown that ChA can increase resistance to pathogens. For example, in chickens, the use of ChA in the diet reduced oxidative stress, inflammation, and small intestine injury induced by *Clostridium perfringens* type A [64]. Some research have shown that the anti-inflammatory properties of ChA might be exerted through inactivation of signaling pathways regulated by IL-8, which is a result of the ROS-scavenging properties of ChA [25]. In addition, the antibacterial properties of ChA were reported by *in vitro* studies [96–100]. It is recognized that ChA may exert antibacterial effects by altering the permeability of bacterial cell membranes [97]. Overall, the antibacterial properties of ChA seem to be related to its destructive effects on the bacterial membrane, which disrupts the enzyme and removes iron, a vital element for bacterial growth [21, 23].

In conclusion, the use of ChA in the fish diet improved growth, digestive enzyme activity, immunity, and resistance to *Y. ruckeri* infection. In addition, ChA showed a protective effect on the liver, as the levels of liver metabolic enzyme decreased in the supplemented fish compared to the control. Finally, a dietary level of 600–800 mg ChA/kg is suggested for the fish, due to its high performance with growth and immunity.

## Figures and Tables

**Table 1:** Feedstuffs and compositions of the basal diet [38].

Ingredients	g/kg	Proximate composition	% in dry basis
Fish meal^a^	310	Crude protein	425
Soybean meal^b^	200	Crude lipid	163
Wheat meal (ChA)	166	Crud ash	79.5
Poultry by-product^c^	130	Moisture	88.2
Wheat gluten^d^	100		
Phytase^e^ (CA)	8		
Fish oil	40		
Lysine^f^	7		
Soybean oil	30		
Methionine^f^	4		
Mineral mix^g^	2.5		
Vitamin mix^g^	2.5		
Total	1000		

^a^Peygir Co., Gorgan, Iran (crude protein 58.8%). ^b^Soyabean Co., Gorgan, Iran (crude protein 45.2%). ^c^Peygir Co., Gorgan, Iran (crude protein 51.0%). ^d^Shahdineh Aran Co. (crude protein 78.3%). ^e^Golbid Co., Tehran, Iran. ^f^Mad Tiour Co., Sanandaj, Iran. ^g^The premix provided following amounts per kg of feed: A: 1000 IU; D3: 5000 IU; E: 20 mg; B5: 100 mg; B2: 20 mg; B6: 20 mg; B1: 20 mg; H: 1 mg; B9: 6 mg; B12: 1 mg; B4: 600 mg; C:50 mg; Mg: 350 mg; Fe: 13 mg; Co: 2.5 mg; Cu: 3 mg; Zn: 60 mg; Se: 0.3 mg; I: 1.5 mg; Mn: 10 mg. Chinechin Co., Tehran, Iran.

**Table 2:** The growth performance of *O. mykiss* fed with different levels of ChA after a 60-day feeding trial.

Parameter	Different experimental diets					
	IW (g)	FW (g)	WG (g)	SGR (%/d)	FCR	SR (%)
Control	18.13 ± 0.08^a^	74.33 ± 0.72^c^	56.20 ± 0.75^c^	2.35 ± 0.01^c^	1.33 ± 0.02^a^	94.00 ± 2.00^a^
CA1	18.16 ± 0.12^a^	79.80 ± 1.13^b^	61.63 ± 1.24^b^	2.46 ± 0.03^b^	1.23 ± 0.03^ab^	94.00 ± 1.00^a^
CA2	18.05 ± 0.11^a^	83.33 ± 1.30^ab^	65.26 ± 1.18^ab^	2.54 ± 0.01^ab^	1.17 ± 0.03^bc^	95.33 ± 2.33^a^
CA3	18.23 ± 0.15^a^	86.43 ± 0.74^a^	68.20 ± 0.78^a^	2.59 ± 0.02^a^	1.08 ± 0.02^c^	97.33 ± 1.33^a^
CA4	18.26 ± 0.14^a^	84.80 ± 0.60^a^	66.53 ± 0.66^a^	2.55 ± 0.02^ab^	1.12 ± 0.01^bc^	93.00 ± 1.73^b^

Data in a column assigned with different letters signify significant difference between treatments (*P* < 0.05). Values are presented as mean ± SE. IW: initial weight; FW: final weight; SGR: specific growth rate; WG: weight gain; FCR: feed conversion rate; SR: survival rate.

**Table 3:** The serum immune parameters of *O. mykiss* fed with different levels of ChA after a 60-day feeding trial.

Parameter	Different experimental diets						
	LYZ (U/mL)	Ig (mg/mL)	C3 (g/dL)	C4 (g/dL)	ACH50 (U/mL)	NBT (540 nm)	MPO (450 nm)
Control	21.94 ± 0.85^c^	15.53 ± 0.52^c^	13.45 ± 0.72^c^	5.43 ± 0.56^c^	113.33 ± 2.60^a^	0.60 ± 0.03^c^	1.26 ± 0.12^b^
CA1	25.40 ± 0.67^bc^	18.57 ± 0.61^bc^	15.10 ± 0.51^bc^	6.83 ± 0.37^bc^	116.23 ± 3.52^a^	0.65 ± 0.03^bc^	1.30 ± 0.05^ab^
CA2	26.97 ± 0.81^ab^	21.03 ± 0.89^ab^	17.76 ± 0.43^ab^	9.36 ± 0.56^ab^	120.33 ± 2.60^a^	0.73 ± 0.02^bc^	1.43 ± 0.04^ab^
CA3	30.19 ± 0.55^a^	23.36 ± 0.69^a^	20.43 ± 0.74^a^	11.30 ± 0.62^a^	123.00 ± 2.30^a^	0.79 ± 0.03^ab^	1.64 ± 0.05^a^
CA4	25.80 ± 0.92^b^	20.53 ± 0.54^ab^	16.70 ± 0.43^b^	8.53 ± 0.63^b^	122.33 ± 2.33^a^	0.91 ± 0.04^a^	1.32 ± 0.07^ab^

Data in a column assigned with different letters signify significant difference between treatments (*P* < 0.05). Values are presented as mean ± SE. LYZ: lysozyme; Ig: total immunoglobulin; C3 and C4: complement components 3 and 4; ACH50: alternative complement activity; NBT: nitroblue tetrazolium; MPO: myeloperoxidase.

**Table 4:** The mucus immune parameters of *O. mykiss* fed with different levels of ChA after a 60-day feeding trial.

Parameter	Different experimental diets				
	LYZ (U/mg pro)	ALP (U/mg pro)	Protease (U/mg pro)	Esterase (U/mg pro)	Ig (mg/mL)
Control	12.08 ± 0.45^b^	26.64 ± 0.39^c^	6.70 ± 0.37^c^	1.83 ± 0.20^b^	11.36 ± 0.44^c^
CA1	13.48 ± 0.61^ab^	28.43 ± 0.69^bc^	8.00 ± 0.34^bc^	2.26 ± 0.14^ab^	12.60 ± 0.49^bc^
CA2	15.17 ± 0.58^a^	29.83 ± 0.44^ab^	10.63 ± 0.55^a^	2.43 ± 0.23^ab^	14.63 ± 0.42^ab^
CA3	15.19 ± 0.60^a^	31.33 ± 0.49^a^	10.36 ± 0.69^a^	2.90 ± 0.15^a^	16.73 ± 0.56^a^
CA4	14.30 ± 0.47^ab^	29.60 ± 0.66^ab^	9.43 ± 0.48^ab^	2.16 ± 0.14^ab^	13.76 ± 0.55^b^

Data in a column assigned with different letters signify significant difference between treatments (*P* < 0.05). Values are presented as mean ± SE. LYZ: lysozyme; ALP: alkaline phosphatase; Ig: total immunoglobulin.

**Table 5:** The serum biochemical parameters of *O. mykiss* fed with different levels of ChA after a 60-day feeding trial.

Parameter	Different experimental diets						
	AST (U/L)	ALP (U/L)	ALT (U/L)	LDH (U/L)	TP (g/dL)	ALB (g/dL)	GLO (g/dL)
Control	151.66 ± 4.40^a^	291.66 ± 4.40^a^	43.00 ± 1.52^a^	831.66 ± 7.26^a^	2.90 ± 0.20^c^	1.80 ± 0.11^a^	1.10 ± 0.15^b^
CA1	132.83 ± 4.18^b^	273.66 ± 3.48^ab^	37.16 ± 1.69^ab^	816.00 ± 8.32^ab^	3.33 ± 0.09^bc^	2.10 ± 0.20^a^	1.23 ± 0.12^ab^
CA2	118.66 ± 1.85^bc^	258.33 ± 6.00^b^	32.00 ± 1.05^bc^	806.00 ± 4.58^abc^	3.76 ± 0.12^ab^	2.46 ± 0.09^a^	1.30 ± 0.11^ab^
CA3	114.33 ± 2.96^c^	232.63 ± 4.33^c^	30.66 ± 1.20^c^	782.66 ± 5.05^c^	4.23 ± 0.24^a^	2.53 ± 0.29^a^	1.70 ± 0.13^a^
CA4	130.33 ± 3.27^bc^	263.70 ± 4.66^b^	34.50 ± 1.32^bc^	800.00 ± 5.77^bc^	3.46 ± 0.15^abc^	1.90 ± 0.06^a^	1.56 ± 0.15^ab^

Data in a column assigned with different letters signify significant difference between treatments (*P* < 0.05). Values are presented as mean ± SE. AST: aspartate transaminase; ALP: alkaline phosphatase; ALT: alanine aminotransferase; LDH: lactate dehydrogenase; TP: total protein; ALB: albumin; GLO: globulin.

**Table 6:** Survival rate and RPS (%) of *O*. *mykiss* fed diets supplemented with different levels of ChA for 60 days, at the end of the challenge with *Y. ruckeri*.

Different experimental diets	SR (%)	RPS (%)
C	35.34 ± 2.00^d^	
CA1	51.16 ± 2.23^c^	24.47 ± 3.45
CA2	64.46 ± 2.23^ab^	45.04 ± 3.45
CA3	75.60 ± 2.20^a^	62.25 ± 3.40
CA4	60.02 ± 3.82^bc^	38.17 ± 5.92

Data in a column assigned with different letters signify significant difference between treatments (*P* < 0.05). Values are presented as mean ± SE. SR: survival rate; RPS: relative percentage of survival.

## Data Availability

The datasets generated during and/or analyzed during the current study are available from the corresponding authors on reasonable request.
